# Biological *vs.* Physical Mixing Effects on Benthic Food Web Dynamics

**DOI:** 10.1371/journal.pone.0018078

**Published:** 2011-03-24

**Authors:** Ulrike Braeckman, Pieter Provoost, Tom Moens, Karline Soetaert, Jack J. Middelburg, Magda Vincx, Jan Vanaverbeke

**Affiliations:** 1 Department of Biology, Marine Biology Section, Ghent University, Ghent, Belgium; 2 Netherlands Institute of Ecology (NIOO-KNAW), Centre for Estuarine and Marine Ecology, Yerseke, The Netherlands; 3 Faculty of Geosciences, Utrecht University, Utrecht, The Netherlands; University of Lancaster, United Kingdom

## Abstract

Biological particle mixing (bioturbation) and solute transfer (bio-irrigation) contribute extensively to ecosystem functioning in sediments where physical mixing is low. Macrobenthos transports oxygen and organic matter deeper into the sediment, thereby likely providing favourable niches to lower trophic levels (i.e., smaller benthic animals such as meiofauna and bacteria) and thus stimulating mineralisation. Whether this biological transport facilitates fresh organic matter assimilation by the metazoan lower part of the food web through niche establishment (i.e., ecosystem engineering) or rather deprives them from food sources, is so far unclear. We investigated the effects of the ecosystem engineers *Lanice conchilega* (bio-irrigator) and *Abra alba* (bioturbator) compared to abiotic physical mixing events on survival and food uptake of nematodes after a simulated phytoplankton bloom. The ^13^C labelled diatom *Skeletonema costatum* was added to 4 treatments: (1) microcosms containing the bioturbator, (2) microcosms containing the bio-irrigator, (3) control microcosms and (4) microcosms with abiotic manual surface mixing. Nematode survival and subsurface peaks in nematode density profiles were most pronounced in the bio-irrigator treatment. However, nematode specific uptake (Δδ^13^C) of the added diatoms was highest in the physical mixing treatment, where macrobenthos was absent and the diatom ^13^C was homogenised. Overall, nematodes fed preferentially on bulk sedimentary organic material rather than the added diatoms. The total C budget (µg C m^−2^), which included TO^13^C remaining in the sediment, respiration, nematode and macrobenthic uptake, highlighted the limited assimilation by the metazoan benthos and the major role of bacterial respiration. In summary, bioturbation and especially bio-irrigation facilitated the lower trophic levels mainly over the long-term through niche establishment. Since the freshly added diatoms represented only a limited food source for nematodes, the macrobenthic effect was more pronounced in niche establishment than the negative structuring effects such as competition.

## Introduction

Phytoplankton blooms are the major source of organic matter for shallow seas like the North Sea. About 20% of the annual phytoplankton bloom settles down to the seafloor as phytodetritus [1]. Shallow benthic communities are generally considered to depend on this input of locally produced organic matter [OM] [2]. OM enters the different parts of the food web, in which grazing macro- and meiobenthos disintegrate and process the larger particles and act in concert with bacteria as key players in mineralisation processes (ammonification, nitrification, denitrification, …). The cycling of this OM is essential to provide the nutrients to sustain primary production [3]. Mixing processes (both physical and biological) at the sea floor play an important role in OM cycling [4]. Intensive physical mixing, induced by e.g. storm events and tidal action [5] dilutes the OM in the surface layer where it was deposited and makes it less accessible to deposit-feeding macrobenthos, but favours bacteria [6] and possibly metazoan members of the lower food web. Biological mixing influences OM availability in two ways: on the one hand, bioturbation and bio-irrigation indirectly alter the distribution of small infauna through establishment of micro-habitats in the otherwise anoxic and food-depleted deep sediment layers [7]–[9]. In addition, dense tube lawns have been found to increase food availability owing to the local decrease in near-bed current velocity [10]. This ecosystem engineering *sensu* Jones et al. [11] contributes extensively to ecosystem functioning in sediments where physical mixing is low [12], [13]. On the other hand, biological mixing goes along with predation or with exploitative competition when the same food source is shared. The effect of biological mixing on infaunal abundance and distribution is well established [7], [14]–[16]. However, the relative importance of the mechanisms through which this effect occurs (food availability, sediment oxygenation), are not fully clear. Whether this biological mixing facilitates the uptake of fresh organic matter by the metazoan members of the lower food web through ecosystem engineering or rather deprives them from food sources, is so far not unambiguously determined.

In a controlled laboratory experiment, we therefore investigated whether phytodetritus uptake by the metazoan members of the lower food web is either facilitated or hampered by biological and physical mixing. Nematodes are an ideal model organism to represent the metazoan lower food web, since they are ubiquitous, numerically the most important metazoans in the biosphere and comprise a high trophic diversity [17]. We added ^13^C labelled diatoms to microcosms containing subtidal sediment with its natural nematode communities but devoid of the natural macrobenthos population. We contrasted a regular physical mixing treatment (upper 2 cm reworked with a sediment stirrer) with the addition of two functionally different macrobenthos species (a bioturbator and a bio-irrigator) in single-species treatments. The two species are dominant representatives of the *Abra alba*–*Kurtiella bidentata* community in the Belgian part of the North Sea [18]. The bio-irrigating polychaete *Lanice conchilega* is a suspension–deposit feeder that lives sedentary with limited impact on particle mixing once the tubes are established. Its piston–pumping [19] induces deep sediment oxygenation and associated stimulation of bacteria as food sources along the tube walls, which results in the extension of the suitable habitat for nematodes [7] and an enhancement of benthic mineralisation [20]. The bioturbating bivalve *Abra alba* is a suspension-deposit feeder that reworks the sediment at random and does not actively irrigate its feeding pits, resulting in a limited stimulation of benthic mineralisation rates [20]. However, its exploitative competition for food at the sediment surface and subsurface faecal pellet deposition has also been shown to extend nematode distribution to deeper layers [7].

We tested whether metazoan lower food web dynamics are (1) affected by mixing; (2) different in biologically or physically mixed sediments; (3) sediment–depth dependent and (4) different for dominant members of the nematode community.

## Results

### Survival of macrobenthos

75±4% of the *Abra alba* specimens were recovered alive at the time of slicing, 17 to 18 days after food addition. The biomass of the bivalves totalled 5.7±0.7 g C m^−2^. *A. alba* individuals were found between 1 and 4 cm of which 89% was concentrated between 1 and 3 cm. *Lanice conchilega* tubes extended to the very bottom of the microcosms (±9 cm) and all animals were alive at the time of slicing. *L. conchilega* biomass totalled 2.1±0.2 g C m^−2^. The total nematode biomass at the end of the experiment averaged 0.020±0.003 g C m^−2^ in the BT treatment and 0.044±0.007 g C m^−2^ in the BI treatment.

### Environmental variables

#### Pigments

**Figure 1:**
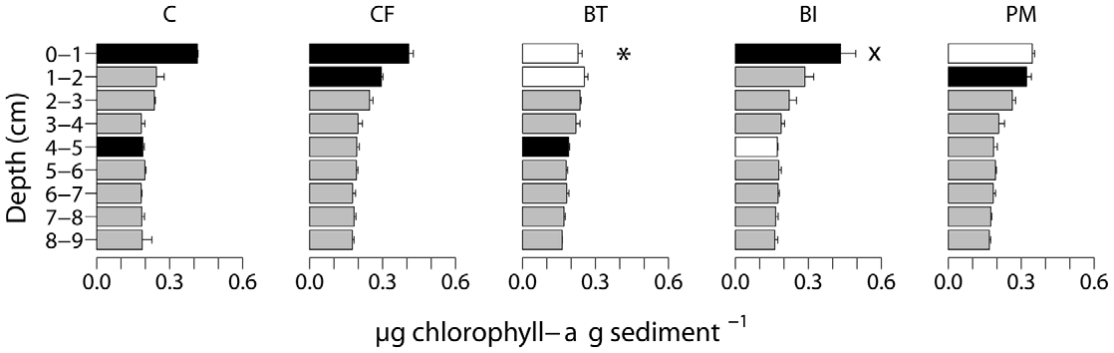
Depth profiles of chlorophyll-a in control (C), control + food (CF), bioturbator (BT), bio-irrigator (BI) and physical mixing (PM) treatments. Profiles differed significantly and significant results of pairwise tests of treatments within TRxD(epth) are indicated by the colour of the bars: black bars indicate higher and white bars lower chlorophyll-a values among pairs; grey bars indicate slices not detected as significantly different from the same depth slices in other treatments by pairwise tests. Significantly lowest values are marked with “*”. “x”: chl-a content in the 0–1 cm of BI treatment only higher than 0–1 cm of BT treatment. Error bars indicate SE.

Total chlorophyll-*a* concentration did not differ among treatments (1–factor Permanova, p>0.05) but its vertical distribution was significantly affected by the different mixing treatments (Table 1): the upper cm of the BT treatments was depleted in chl-*a* compared with the same layers in the other treatments. Even the second cm of the BT treatment contained less chl-*a* than the same one in the PM and CF treatment. Furthermore, the 4–5 cm layer from the C and BT treatment had a significantly higher concentration than that of the BI 4–5 cm (Table S1, Fig. 1). Compared to the C treatment, the chl-*a* profile was less steep in the CF, BI and BT treatment (being depleted in the upper cm of the BT treatment) and this photopigment was homogenised over the upper 2 cm in the PM treatment (Table S2).

**Table 1:** Results from Permanova analysis for differences in chlorophyll-*a* content (µg g^−1^), total TO^13^C (mg m^−2^) within the sediment and nematode density (ind. 10 cm^−2^) amongst experimental treatments (TR) (control, control+food, bioturbator, bio-irrigator and physical mixing) and depth (D), based on a Euclidean resemblance matrix.

*Factors*		*Chlorophyll-a*	*TO^13^C sediment*	*Nematode density*
TR	df	4	3	4
	MS	3.23E-03	55.92	2589.5
	Pseudo-F	3.76	**6.12** a	**10.10** a
D	df	8	8	8
	MS	5.74E-02	559.72	9739.8
	Pseudo-F	**108.64** b	**117.75** b	**35.83** b
Rep (TR)	df	9	8	9
	MS	8.58E-04	9.14	0.943
	Pseudo-F	1.62	1.92	2.55
TRxD	df	32	24	32
	MS	2.73E-03	32.45	692.73
	Pseudo-F	**5.16** b	**6.83** b	**2.55** a
Res	df	72	64	72
= Rep(TR)xD	MS	5.28E-04	4.75	271.87

a : 0.001 <p<0.05;

b : p<0.001

Control was not included in the TO^13^C sediment analysis.

#### Sediment organic matter content

**Figure 2:**
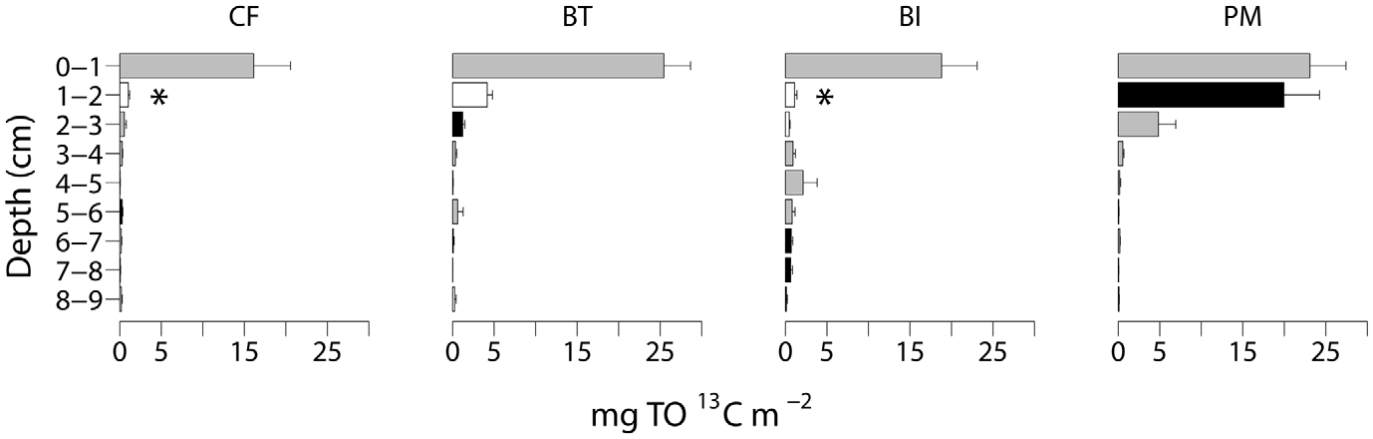
Sediment TO^13^C distribution in control + food (CF), bioturbator (BT), bio-irrigator (BI) and physical mixing (PM) treatments. Profiles differed significantly and significant results of pairwise tests of TR within TRxD are indicated by the colour of the bars (*cf.* Figure 1). Significantly lowest values are marked with “*”. Compare with the original addition of ±400 mg algal ^13^C to the surface of the microcosms at the start of the experiment. Error bars indicate SE.

About 10% of the initially added TO^13^C remained in the sediment at the end of the experiment and the remnants significantly varied among treatments (Table 1). In the end, TO^13^C concentrations were highest in the BT treatment (pairwise test, p(MC)  = 0.041) and the PM (pairwise test, p(MC)  = 0.032) treatment compared to the CF. The leftovers of the labelled algae showed different vertical distributions among treatments (Table 1, Fig. 2): they mainly remained at the surface in the CF treatment, whereas they were efficiently mixed downwards to 3 cm depth in the PM treatment and to a lesser extent in the BT treatment (Table S3, S4). At depth of the BI treatment (6–9 cm), small TO^13^C concentrations were measured. Hardly any trace of label was found at depth of the CF and PM treatment.

#### Oxygen penetration depth and consumption

**Figure 3:**
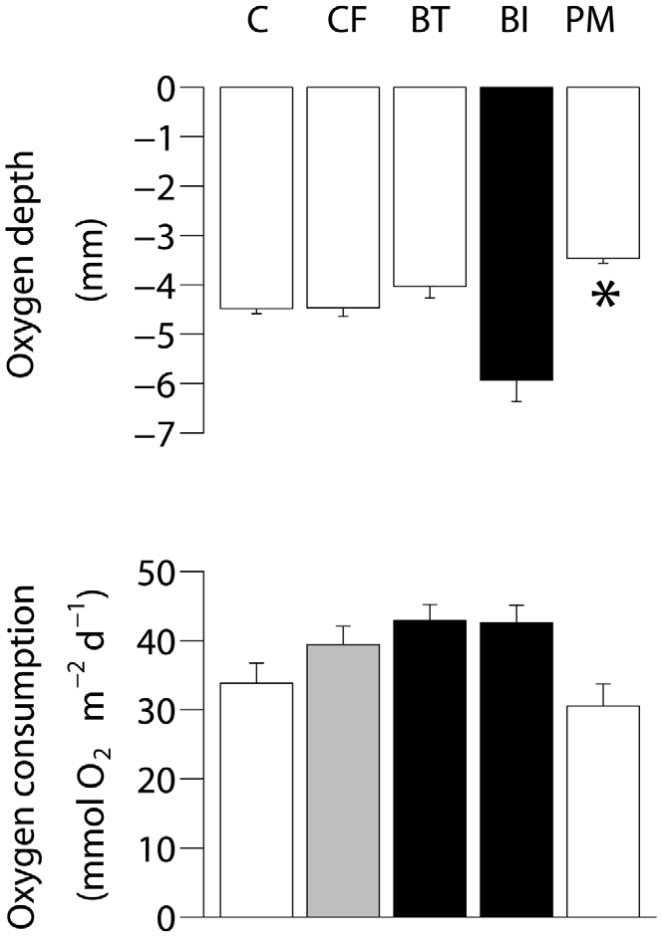
Maximum oxygen depth at the end of the experiment (upper) and averaged oxygen consumption of measurement on day 3 and 10 (lower) in control (C), control + food (CF), bioturbator (BT), bio-irrigator (BI) and physical mixing (PM) treatments. Results of pairwise tests are indicated by the colour of the bars (*cf.* Figure 1). Significantly lowest values are marked with “*”. Error bars indicate SE.

Oxygen penetration reached an average depth of 4.5±0.2 mm, but differed among treatments (Permanova pseudo-F_4,70_ = 14.18, p = 0.001) and was significantly deeper in the BI microcosms (5.9±0.4 mm) and significantly shallower in the PM treatment (3.5±0.1 mm) (Fig. 3, pairwise tests Table S5). There were no significant differences for the TRxDay interaction (2-way Permanova, p = 0.80) in oxygen consumption, but differences between treatments and between days were observed (TR: pseudo-F_4,20_ = 4.72, p = 0.008; Day: pseudo-F_1,20_ = 5.98, p = 0.024): sediment community oxygen consumption (SCOC) was lower in the PM and C treatment compared to both the BT treatment (pairwise tests BT-C: p(MC)  = 0.016; BT–PM: p(MC)  = 0.008) and BI treatment (BI–C: p(MC)  = 0.041; BI–PM: p(MC) = 0.019). SCOC measurements were highest on day 10, but are for simplification presented as averages of the two measurement days in Fig. 3.

### Biota

#### Nematode distribution

**Figure 4:**
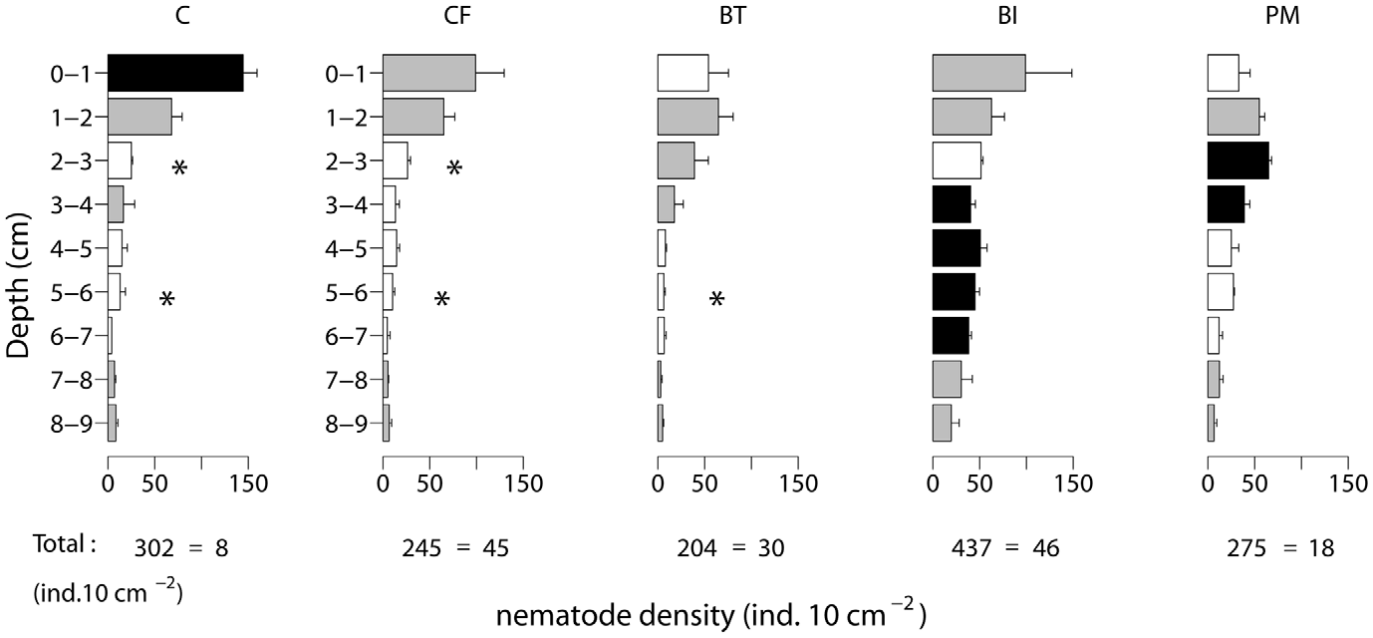
Depth profiles of nematode densities in control (C), control + food (CF), bioturbator (BT), bio-irrigator (BI) and physical mixing (PM) treatments. Profiles differed significantly and significant results of pairwise tests of TR within TRxD are indicated by the colour of the bars (*cf.* Figure 1). Significantly lowest values are marked with “*”. Error bars indicate SE

Total nematode density differed among experimental treatments (Fig. 4, Table 1) and was highest in the BI microcosms (pairwise tests, p(MC) <0.05 for comparisons with CF, BT and PM treatments). The survival in the BT treatment was significantly lower than that in the PM (pairwise test p(MC)  = 0.038). Nematode density profiles differed among treatments (Table 1, Fig. 4): most nematodes concentrated in the top layer in the C, CF and BI treatment (although with a high variability among replicates in the latter two) and their density declined rapidly below 2 cm in the C and CF treatment, while it was more or less equally distributed in the BI treatment (Table S7). Nematodes in the PM treatment shifted to the undisturbed subsurface layers (below 2 cm). Nematode density in the deeper layers of the BI treatment was higher than in the deep layers of the other treatments (Table S6, Fig. 4).

#### Nematode food uptake

**Figure 5:**
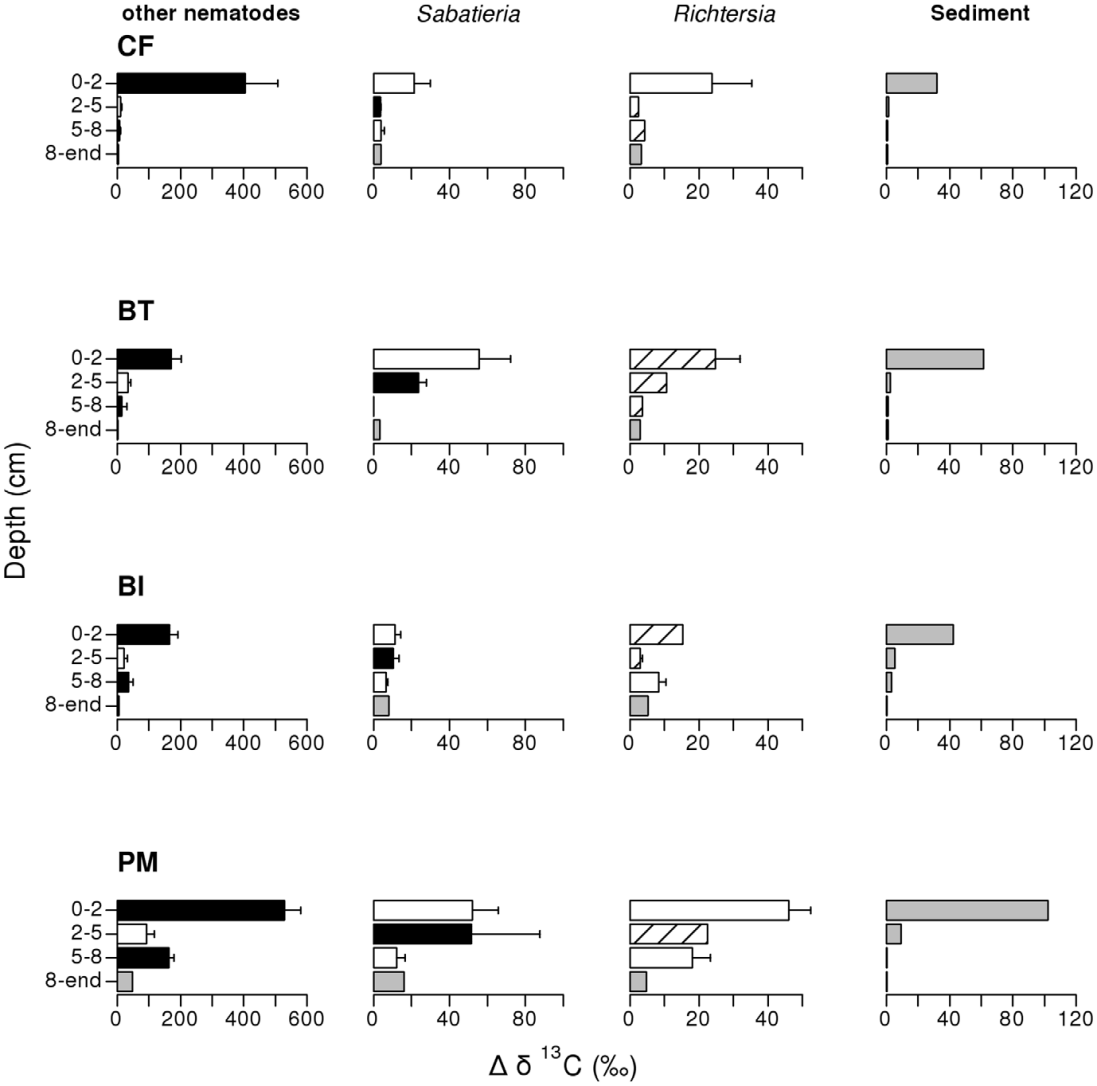
Specific uptake (Δδ^13^C) of labelled diatoms by ‘other nematodes’, *Sabatieria* and *Richtersia* and averaged TOC in the different depth layers in the control + food (CF), bioturbator (BT), bio-irrigator (BI) and physical mixing (PM) treatments. Specific uptake differed among TR (PM> BI and BT) and among DxSp. For nematodes, the results of the pairwise tests of Sp within DxSp interaction are indicated by the colour of the bars (*cf.* Figure 1). Coarse striped bars indicate <3 replicates. Note different scaling of x-axes. Error bars indicate SE.

Nematode samples showed a varying degree of ^13^C enrichment. Nematode specific uptake (Δδ^13^C) ranged between 1.6 (‘other nematodes’ in the 8-end layer of the BT treatment) and 864 ‰ (‘other nematodes’ in 0–2 cm of the CF treatment) and was different among TR and among Species and Depths (SpxD interaction and TR factor significant, Table 2, Fig. 5). Nematodes showed the highest specific uptake in the PM treatment compared to the BT (pairwise test: p(MC) = 0.022) and BI treatment (p(MC)  = 0.018) and this specific uptake in the PM treatment was slightly higher than in the CF (p(MC)  = 0.081) treatment. At intermediate depths, *Sabatieria* had a higher specific uptake than the ‘other nematodes’ (p(MC)  = 0.017) and *Richtersia* (p(MC) <0.001). In the deeper layer, ‘other nematodes’ showed a higher specific uptake than the other two groups (p(MC)  = 0.002). These ‘other nematodes’ showed a particularly high specific uptake in the 5–8 cm of the PM treatment although no traces of ^13^C were found in the sediment at that depth. The nematode community in this layer was mainly composed of *Sabatieria breviseta* (36%) and *S. celtica* (15%), with *Daptonema* spp. (7%) and *Trefusia* spp. (7%) as members of the ‘other nematodes’. At the surface, ‘other nematodes’ showed a higher specific uptake than *Sabatieria* (pairwise test: p(MC)  = 0.0001) and *Richtersia* (p(MC)  = 0.0003). For ‘other nematodes’, the SF-value (% of labelled algae in food taken in excess of bulk OM), calculated from the δ^13^C signal in nematodes, diatoms and in averaged sediment TOC (in Fig. 5) was very small, ranging between 0.24% (BT treatment) and 0.84% (PM treatment) in the surface layer. In the case of *Sabatieria* and *Richtersia*, the SF-value at the surface of the CF and BI treatment was even slightly negative, indicating that the δ^13^C in the sediment was higher than the δ^13^C in the nematodes. Although the SF-ratio was always >0 in the subsurface layers, the selectivity for the labelled diatoms remained very limited, never exceeding 1%.

**Table 2:** Results from Permanova analysis for differences in Δδ^13^C (‰) and total uptake I_total_ (µg C m^−2^) amongst the experimental treatments (TR), replicates (Rep) nested in TR, depth layers (D) and nematode groups (Sp) and their interactions TRxD, TRxSp, DxSp, Rep(TR)xD, Rep(TR)xSp, Rep(TR)xDxSp, TRxDxSp and Error term, based on a Euclidean distance based resemblance matrix (df = 3,3,2,8,9,6,6,15,15,15,17,48 resp.).

*Factor*		*Δδ^13^C*	*I_total_*
TR	MS	17787	57.14
	pseudo-F	**4.33** a	0.34
D	MS	55818	697.08
	pseudo-F	**12.89** c	2.49
Sp	MS	64375	323.94
	pseudo-F	**11.91** b	1.53
Rep (TR)	MS	1135.8	111.82
	pseudo-F	9.85E-02	0.37
TRxD	MS	4703.6	81.25
	pseudo-F	1.25	0.29
DxSp	MS	66871	1031.6
	pseudo-F	**19.28** c	**4.03** a
Rep(TR)xD*	MS	1870.5	271.5
	pseudo-F	0.16	0.89
Rep(TR)xSp*	MS	2135	160.97
	pseudo-F	1.19	0.53
TRxSp	MS	10258	43.60
	pseudo-F	1.95	0.21
Rep(TR)xDxSp*	MS	1917.3	246.28
	pseudo-F	0.16	0.81
TRxDxSp*	MS	7332.1	130.86
	pseudo-F	2.15	0.51
Res	MS	11535	305.88

a *: 0.01< p<0.05;*

b *: 0.001<p<0.01;*

c *: p<0.001*

“*”: term has one or more cells with a single replicate.

**Figure 6:**
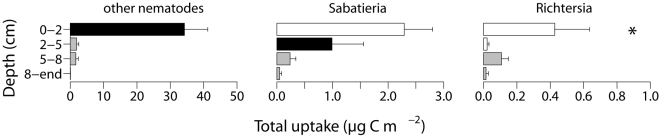
Total uptake (µg C m^−2^) of labelled diatoms by ‘other nematodes’, *Sabatieria* and *Richtersia* in the different depth layers. Total uptake only differed among DxSp. The results of the pairwise tests of Sp within the DxSp interaction are indicated by the colour of the bars (*cf.* Figure 1). Significantly lowest values are marked with “*”. Error bars indicate SE.

Total uptake did not differ significantly among treatments, probably due to the high variance in uptake and nematode densities in the surface layers of the CF and BI treatments (Fig. 4). However, the nematode groups did differ in their uptake of diatom carbon within depth layers (Table 2, Fig. 6). At the surface, the total uptake of ‘other nematodes’ was higher than the uptake by *Sabatieria*, which in turn was larger than the uptake by *Richtersia* (virtually zero). In the intermediate depth layer, the *Sabatieria* uptake was higher than that of *Richtersia*. The total uptake by *Richtersia* did not differ among depth layers.

The total uptake as a percentage of the added diatom carbon by the nematodes integrated over depth ranged between 0.0024±0.0008% (BT treatment) and 0.0058±0.0027% (CF treatment), with intermediate values in the PM (0.0042±0.0008%) and in the BI treatment (0.0039±0.0029%).

#### Macrobenthic food uptake

The diatom addition triggered the immediate response of *Abra alba* and *Lanice conchilega*, as apparent from the sudden appearance of the siphons and tentacles at the surface and their instant clearing of the diatom mat. As a result, the average specific uptake of the macrobenthic animals after 17/18 days was quite high: 117±66‰ in *A. alba* and 256±43‰ in *L. conchilega*. In terms of total uptake, the consumption of the enriched diatoms by *A. alba* and *L. conchilega* tissue totalled respectively 2.5±0.6% and 2.7±0.9% of the added diatom carbon after 18 days. This is about 3 orders of magnitude higher than the total uptake by nematodes, but is partly explained by the 100x higher macrobenthic biomass.

### Budget

**Figure 7:**
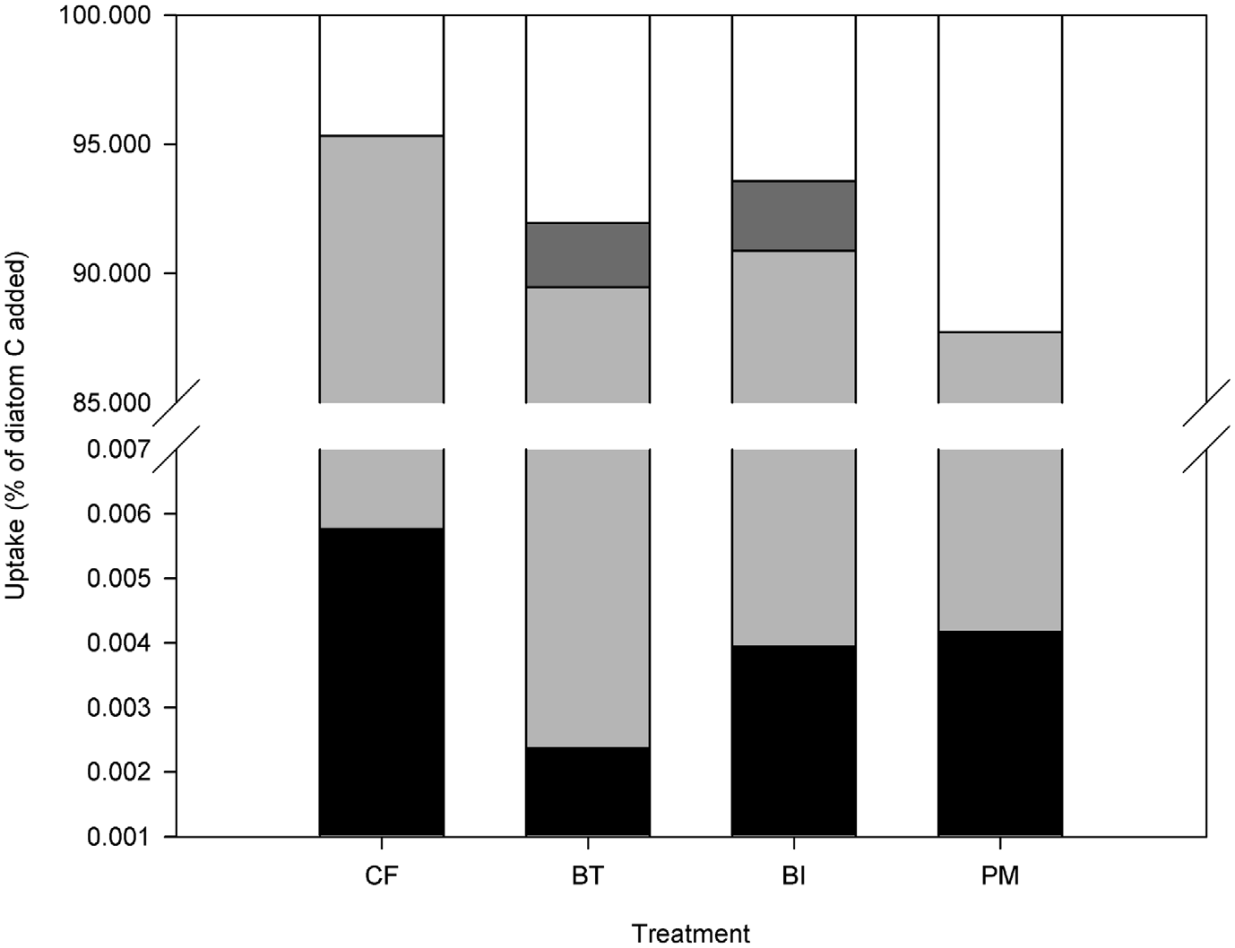
Mineralisation of the added diatom C in the control + food (CF), bioturbator (BT), bio-irrigator (BI) and physical mixing (PM) treatment by the nematode (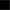, black) and macrobenthic (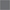, gray) compartments and due to respiration (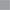, light gray). Only “Remaining” algal C (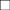, white) differs significantly among treatments. Error bars not shown.

Fig. 7 shows the contribution of the different compartments to the fate of the added diatoms. After 18 days, <3% of the added diatom ^13^C was assimilated by the macrobenthos and nematode compartment, about 10% was left in the sediment and the rest (±90%) can be assumed to have been respired. From the increase in SCOC between the C and the CF treatment (on average 5.60 mmol O_2_ m^−2^ d^−1^) (Fig. 3), it can be estimated (assuming a respiratory quotient of 0.85 [21]) that 1.03 g C m^−2^ was respired over the 18 days of experiment, equalling 104% of the added carbon.

## Discussion

Mixing processes are important for degradation of phytodetritus [22] and bacterial members of the lower food web: in case phytodetritus arrives at the surface and remains unmixed, only bacteria in the top layer can profit from the deposition event, whereas bioturbation and bio-irrigation can supply food to deeper living bacteria [6]. The effect of mixing processes on the dynamics of the metazoan members of the lower food web was so far unknown. In this experiment, both physical and biological mixing had a pronounced and contrasting effect on the dynamics of the metazoan members of the lower food web. Biological and physical mixing both induced reallocation of this smaller infauna to the subsurface compared to the control situation. Although survival of this smaller infauna was highest in the bio-irrigated treatment, this did not coincide with the highest diatom uptake in smaller infauna. In contrast, physical mixing induced the highest access to food by the smaller infauna over 17/18 days.

### Mixing effects on environmental parameters

While chl-*a* and TO^13^C - indicators of the freshly added diatoms - remained at the surface of the control treatments, a clearly different environment was created in the microcosms experiencing physical and biological mixing. Chl-*a* was depleted in the upper 2 cm of the bioturbation treatment, revealing intense grazing by the bivalve [4], [7]. In the subsurface (2–3 cm) of the bioturbation microcosms, the labelled material accumulated, which might be in the form of faecal pellets [23]. High concentrations of chl-*a* were also found at the surface of the bio-irrigated microcosms, while small peaks of TO^13^C were traced at depth (6–9 cm). At the same time, oxygen was drawn deeper into the sediment owing to bio-irrigation. Physical mixing efficiently distributed chl-*a* and TO^13^C over the upper two centimetres and oxygen penetration was shallower and SCOC lower than in the other treatments. Since macrobenthos was absent, transport occurred mainly via molecular diffusion, resulting in a lower oxygen flux from water to sediment (SCOC). Initial deep oxygen penetration was also observed in other physical mixing experiments [22], but the redox boundary then rapidly shifted to shallower depths and the buried OM degraded at a slower pace via anaerobic pathways [22]. The significantly higher TO^13^C pool remaining in the physically mixed microcosms at the end of the experiment also corroborates the hypothesis that degradation is slower when organic matter is buried [22]. Similarly in the bioturbated microcosms, organic matter was taken away from the faster aerobic degradation processes at the surface because *Abra alba* filters large amounts of water resulting in subsurface deposition of its faecal pellets [23].

### Mixing effects on lower food web dynamics

#### Distribution

Nematode survival was enhanced in the bio-irrigated treatment. The presence of bio-irrigators induces an extension of the habitat of smaller infauna from the surface into the deeper sediment horizons and this appears to be important for the survival of the lower metazoans [7], [24], [8]. In the control treatment, nematodes remained at the surface, while they redistributed over the upper 3 cm in the bioturbation treatment. This subsurface concentration of nematodes is probably explained by a combination of competition for food at the surface (see further), disturbance by the bivalve siphons and attraction to faecal pellets deposited in the subsurface [7]. In the bio-irrigation treatment, oxygen is drawn deeper into the sediment, which is directly beneficial to oxiphilic nematodes [7]; indirectly this oxygen in the deep sediment layers stimulates the growth of bacteria in the tube mucus linings [25] and/or in the sediment surrounding the tubes [26], providing food for the nematodes. In contrast, nematodes in the physically mixed treatment concentrated in the 2–4 cm horizon, which may indicate intolerance to disturbance at the surface or habitat creation at depth (burial of organic matter as a food source).

#### Food uptake

The freshly added diatoms were most accessible (highest specific uptake) to the nematodes in absence of biological mixing. The slightly higher specific uptake in the physically mixed treatment compared to CF suggests that diatom consumption was easier for the nematodes when it was diluted over the 2 cm compared to the concentrated layer in the CF treatment as has been reported for bacteria [6].

Specific uptake by the nematodes at the *surface* of the biologically mixed treatments was only 20–50% of that at the surface of physically mixed treatments, except for *Sabatieria* (equal specific uptake at surface in BT and PM treatments). This indicates that macrobenthos was favoured over the nematodes in terms of access to the diatoms in the concentrated patch [6] (exploitative competition). However, macrobenthos assimilation was low as well (<3% of the added diatom C incorporated after 18d). This indicates that we probably missed the immediate assimilation of the added algae [6], [27]–[29]. Indeed, the sudden appearance of siphons and tentacles at the surface indicates a fast and may be one-off feeding response that may have been missed after 18d due to the rapid turn-over of assimilated algal matter (starting after 5 days [30]). Although this turn-over accounts for nematodes as well, it is possible that they could continue feeding on the diluted patches of labelled matter [6] until the end of the experiment. Alternatively, part of the ^13^C assimilation by nematodes may have resulted from feeding on bacteria which had utilised organic matter originating from the labelled diatoms [31].

In the *deeper* layers, nematode uptake was again highest in the physically mixed treatment. The uptake in this deep layer (5–8 cm) of the physically mixed treatment is even similar to the uptake at the surface of the biologically mixed treatments, although no label was measured in the bulk sediment, deeper than 3 cm. This indicates active migration of nematodes, either by surface dwelling nematodes that migrated to depth at day 1 to avoid disturbance (first physical mixing event after food addition); or deep dwelling nematodes moving to the surface to feed and then returning to deeper layers. The genera found in this depth layer (*Sabatieria celtica* and *S. breviseta*, *Daptonema* spp. and *Trefusia* spp.) have a long and slender body type, which favours migration and anoxia resistance [32]. The low δ^13^C signal of *Sabatieria* and *Richtersia* at the surface of the CF and BI treatments rather reflects the sedimentary δ^13^C signal of the deeper layers, which suggests migration between deeper and surface layers. For *Sabatieria* spp. [33]–[35] and *Daptonema* spp. [36] this migration to fresh food had been observed already, but this is the first time for *Trefusia* and *Richtersia* spp.

A small uptake at depth in the biologically mixed treatments was observed, primarily in the ‘other nematodes group’. This might be related to the transport of label (Fig. 2) and thus food sources to depth [27]. Rapid subduction of labelled ^13^C has been shown frequently both in deep sea environments [29], [8] and North Sea sediments [27]. In our study, this subduction is not pronounced: a similar density of tube-irrigating polychaetes subducted 6 to 23% of the added label deeper than 5 cm [8], while *Lanice conchilega* was responsible for the burial of only 0.6% of the TO^13^C deeper than 5 cm (calculated from Fig. 2). Similarly, Tellinid bivalves in [27] at half the density of *Abra alba* in this experiment showed a strong bioturbation capacity that resulted in increased ΔδTO^13^C values in the 4–7 cm sediment layer of an order of magnitude higher than those found in the bioturbator microcosms. Nevertheless, this small amount of subducted algal material and eventually stimulated bacteria that feed on the diatom ^13^C resulted in labelled nematodes at depth. Alternatively, the nematodes could also have migrated from the surface to depth to avoid disturbance and competition or to take profit from the newly created habitat at depth of the biologically mixed treatments.

Specific uptake of the added diatoms differed among nematode groups and depth layers. Generally, nematodes are analysed in bulk (because of practical reasons, biomass constraints, high genus diversity), which dilutes the uptake signals, since the response of a variety of feeding types is pooled. Here, we were able to differentiate the uptake of 2 dominant genera from the uptake of the ‘other nematodes’ pool. The uptake rates and the index of selectivity (SF-value) were higher in ‘other nematodes’ than in *Sabatieria* and *Richtersia*, probably because the latter migrated more often in between surface (enriched) and deeper (non-enriched) layers, hence a mixed signal in their diet. *Sabatieria* and *Richtersia* demonstrated a slightly negative SF-value in the surface layers of the CF and BI treatments, which contrasts with a positive SF-value in the more mixed BT and PM treatments. This suggests that these migrating nematode groups were also feeding non-selectively on the bulk organic matter and on diatoms in case these were mixed into the bulk organic matter (BT and PM treatments). However, the total uptake and selectivity were very low (SF-ratio's <1% in all cases).

#### Budget

The low total uptake of fresh labelled food by nematodes even after 17/18 days is not unusual [33], [37], [38]. Since this uptake does not meet the nematodes daily respiratory needs (0.66% in [33], 0.1–5.1% in [38]), it is clear that meiobenthos does not feed exclusively on the fresh algal material added to the sediment microcosms, but also exploits other carbon sources, naturally available in the sediment microcosms [39], [33]. This is indeed consistent with the low SF-value, indicating non-selective feeding. At the end of the experiment, only between 4.7 and 12.3% of the added label was recovered as TO^13^C in the sediment, while <3% of the label had accumulated in the fauna. The difference in SCOC between the control with and without food was in the range of the amount of added diatoms (104%), taking into account measurement and conversion errors. SCOC comprises respiration of macro – and meiofauna (generally max. 20%) and bacteria. This points at the large role of bacteria in the mineralisation of the added algal matter [29], [40].

### Conclusions

We have shown that the metazoan members of the lower food web feed non-selectively on the bulk organic matter. Thus, after a pulse deposition of fresh diatoms, they rely on external processes to mix the food in the sediment matrix. Physical mixing diluted the diatom layer into the subsurface, which increased its accessibility for the metazoan members of the lower food web, but slowed down the bacterial degradation as reflected in oxygen consumption. Bioturbation, and especially bio-irrigation facilitates the lower trophic levels both on the short-term (supply of phytodetritus to bacteria [6]) and the long-term (deep density peaks and enhanced survival of small infauna in this experiment). Since the added diatoms contributed only to a limited extent to the carbon requirements of the metazoan members of the lower food web, this macrobenthic facilitation via transport processes was more important in structuring the lower trophic levels than were negative effects such as competition. The present study considered the effects of bioturbators and bio-irrigators at average natural densities in microcosms. However, high density patches of the studied species often occur in the field [41]–[44]. It has been shown that the ecosystem engineering effects of the bio-irrigating polychaete *Lanice conchilega* are density dependent, reaching a maximum at a certain threshold density (>1500 ind.m^−2^), after which the facilitation effects may decline [20], [42], [43]. Similar patterns can be expected in high density patches of the bioturbating bivalve *Abra alba*.

## Materials and Methods

### Study site and sampling

Sediment from a fine sandy coastal station (known as Stn 115bis; 51° 09.2′ N, 2°37.2′ E; 3.5 km off the coast, 13 m depth) was collected with a NIOZ Boxcorer from the *RV* Belgica. Characteristics of the benthic communities at Stn 115bis are given in [45] and [39]. Sediment median grain size was 194.6±1.1 µm, sediments comprised 4.2±0.5% silt and 0.09±0.003% organic carbon. The sediment was sliced into 0–1 cm, 1–3 cm, 3–5 cm, 5–8 cm, 8-end cm sections, wet sieved to remove macrobenthos (>1 mm) and finally brought to a temperature-controlled room in the lab (14°C) at day -9. The water overlying the 0–1 cm section was aerated overnight to ensure survival of oxygen-sensitive nematodes in that layer. At day -8, the sediment column was reconstructed by stacking subsequent sediment horizons in cylindrical microcosms of 14.5 cm internal diameter and 30 cm height. These microcosms were left to acclimatise in the dark for 24 h at 18°C with recirculating filtered seawater (15 cm) of salinity 32.

The bio-irrigating polychaete *Lanice conchilega* was collected in the intertidal area by means of metal frames [46] at day -15, left to acclimatise for 8 days within its natural sediment and subsequently introduced into the microcosms within its tube as described in [47] at a density of 850 ind.m^−2^ (14 ind. microcosm^−1^). To check the fitness of the animals, the fringed tube end was cut after introduction to the sediment. The next day, all animals had rebuilt a new crown of fringes. The bioturbating bivalve *Abra alba* was sampled at day -3 with a Van Veen grab from the *RV* Zeeleeuw and introduced into the microcosms at a density of 1273 ind.m^−2^ (21 ind. microcosm^−1^), which is within the ranges of its natural density [48]. *A. alba* specimens that did not burrow within 30 min. were replaced by fitter individuals.

### Experimental set-up

15 microcosms were allocated to 5 treatments in triplicate: 2 controls (reconstructed sediment without additional macrobenthos; hereafter referred to as experimental control [C] and reconstructed sediment without additional macrobenthos, however with food added, hereafter referred to as [CF]), sediments with the bioturbator *Abra alba* added, sediments with the bio-irrigator *Lanice conchilega* added and sediments receiving physical mixing (hereafter referred to as [BT], [BI] and [PM] treatment). In the latter, the sediment was physically mixed by a simple device manually stirring the sediment for 30 s to a depth of 2 cm starting from day -1 and this was repeated every 48 h. Microcosms were incubated at constant room temperature of 18°C for 18 days. We have deliberately chosen this experiment duration based on observations of maximal meio – and macrobenthic uptake [30], [33]. Microcosms were fitted with a lid equipped with a magnetic stirrer and water was continuously aerated and recirculated at a rate of 14.4 l h^−1^ to an aerated tank with 180 l of filtered seawater at salinity 32.

### Culturing and addition of algae

A strain (radius: 3.8 µm) from the diatom *Skeletonema costatum*, which naturally occurs during the autumn bloom in Belgian coastal waters [49] was obtained from the NIOO-CEME and cultured axenically in f2 medium [50] in sterile Erlenmeyer flasks at 16°C with a 12:12 h light-dark period. Diatoms were labelled through addition of NaH^13^CO_3_ (^13^C, 99%, Cambridge Isotope Laboratories, 336 mg per 100 ml milliQ H_2_O). After labelling, the medium was removed and the diatom cells were harvested by triple centrifugation (3500 rpm, 5 min), lyophilised and stored at −80°C prior to experimental use. This labelling technique resulted in an average δ^13^C value of 59 776 ‰ equalling ca. 40.46% algal ^13^C. On day 0 the frozen algae were thawed and 2.94 ml of diatom suspension was added to each microcosm by means of a long pipette. This equals a concentration of 986.46 mg C m^−2^ (383.73 mg ^13^C m^−2^), which is in accordance with the magnitude of the natural phytoplankton deposition during the entire autumn bloom [1]. Magnetic stirrers attached to the microcosm lids were switched off during diatom addition and only turned on again 1 h after complete settlement of the diatoms. The algal carbon deposition resulted in an increase of sediment organic carbon concentration of 27% of the carbon in the upper cm of the CF treatment and of 14% carbon over the entire CF treatment compared to the C treatment.

### Oxygen depth and consumption measurements

The flux of oxygen across the sediment-water interface was determined on day 3 and 10. Each core was sealed with an air-tight lid and incubated in darkness for max. 6 h, which prevented the overlying water oxygen concentration to decrease below 50% air-saturation. O_2_ concentration was measured with a Unisense microsensor (type ox25) in start and end samples (10 ml) of bottom water and O_2_ consumption was then calculated assuming a linear decrease in O_2_ concentration. Vertical oxygen profiles (5 per microcosm) were measured at the end of the experiment (day 16) using Unisense oxygen microsensors (type ox25) in vertical increments of 250 µm.

### Slicing

On day 17 and 18, all experimental microcosms were sliced in 1 cm sections. The sediment slices were homogenised and subsamples were taken for nematode density and community analysis (10 ml) and stored in a buffered 4% formalin solution, stained with Rose Bengal. Meiofauna was extracted by centrifugation with ludox [17]. All nematodes were counted and the samples from the deep layers of the physical mixing treatment were hand-picked, mounted onto slides and identified to genus or species level where possible according to the pictorial key of [51] and using the NEMYS database [52]. The remaining sediment in each slice was further subsampled for analyses of Total Organic Carbon [TOC] (2 ml), pigments (2 ml) and nematodes for stable isotope analysis (remaining, approx. 180 ml). TOC and pigment subsamples were stored at −80°C until analysis. Next, pigment samples were lyophilised and pigments were extracted in 90% acetone from the homogenised sample. Chlorophyll-*a* [chl-*a*] concentrations of the supernatant were determined using HPLC (Gilson) analysis [53]. TOC samples were lyophilised, homogenised and acidified with dilute HCl until complete decarbonisation. After acidification, TOC was measured using a Thermo Scientific Flash 2000 elemental analyser.

### Isotope analysis, data treatment and analysis

Since slicing the microcosms often implied sacrificing the macrobenthic species, gut clearance was not possible. Sand tubes and bivalve shells were removed and the entire macrobenthic tissue was freeze-dried, ground and a subsample of 0.5 g was prepared in pre-glown (overnight at 550°C to remove any contaminating OM) Ag cups for isotope analysis. For sediment isotope analysis, aliquots of 0.5 g without conspicuous fauna were dried, ground and homogenised. This sediment was then acidified in Ag cups with dilute HCl to eliminate the carbonate fraction. The cups were subsequently pinch closed and stored in Multi-well Microtitre plates under dry atmospheric conditions until analysis.

For stable isotope analysis of nematodes, sediment from within each replicate, but from several slices was combined in 0–2 cm, 2–5 cm, 5–8 cm and 8-end cm to gather sufficient nematode biomass. Frozen natural and enriched nematode samples were thawed, rinsed over a 38 µm sieve and centrifuged once with Ludox to extract the meiofauna. The colloidal silica gel Ludox does not affect the δ^13^C signal of the nematodes as observed during laboratory tests (Moens, unpubl. data). No colouring or preservatives were used to avoid C contamination of the samples. After centrifugation, the nematodes were washed with milliQ water and hand-picked immediately with a fine sterile needle, rinsed in milliQ water to remove adhering particles and transferred to a drop of milliQ water in 2.5×6 mm pre-glown (550°C) Al cups. Nematodes from the dominant genera *Sabatieria* and *Richtersia* (hereafter referred to as ‘*Sabatieria*’ and ‘*Richtersia*’) were picked separately from other nematodes (hereafter referred as ‘other nematodes’). The cups were oven-dried at 60°C, pinched closed and subsequently stored in Multi-well Microtitre plates under dry atmospheric conditions until analysis. Lipids were not extracted from the macrobenthic and nematode samples. The macrobenthos, nematodes and sediment were analysed for δ^13^C_org_ using a Fisons CN elemental analyzer coupled online, via a Finnigan Conflo Il interface, with a THERMO Finnigan Mat Delta Plus Isotope Ratio Mass Spectrometer. Data are expressed in standard δ-unit notation, where δ^13^C = [(R_sample_/R_VPDB_) − 1] ×10^3^, where R is the ^13^C:^12^C ratio. These values are reported relative to the Vienna Pee Dee Belemnite standard (VPDB) with an isotopic ratio of R_VPDB_ = 0.0111797. Incorporation of ^13^C is reflected as excess (above background) ^13^C and is expressed as specific uptake (Δδ^13^C, the difference between δ^13^C of the enriched sample and the natural δ^13^C value of the non-enriched sample) and total uptake in µg ^13^C m^−2^ calculated as the product of excess ^13^C (E) and total biomass (organic carbon) [54] and further expressed in terms of C by correcting for the algal labelling (40.46%). For TO^13^C, the bulk uptake is calculated as the product of excess ^13^C, the organic C content, the porosity and the density (assumed to be 2.55 g cm^−3^). E is the difference between the fraction ^13^C of the sample (F_sample_) and the control (F_reference_), where F = ^13^C/(^13^C+^12^C) = R/(R+1). The carbon isotope ratio (R) was derived from the measured δ^13^ C values as R =  (δ^13^C/1000+1) × R_VPDB_.

If we assume that nematodes feed on the one hand on bulk sediment (isotopic ratio δTO^13^C sediment) and on the other hand specifically select labelled diatoms (δ^13^C diatoms), and assuming that the isotopic composition of nematodes is a weighted fraction of its food sources, we have:
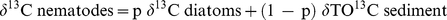
where p is the fraction of algae selectively ingested.

Based on this equation we calculated an index of selective feeding (SF  =  p*100):



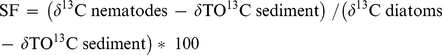
where δ^13^C is about 15–20‰ lower than the Δδ^13^C shown in Fig. 5 (δ^13^C of non-enriched nematode and sediment samples ≈−15 to −20‰). SF was calculated for the different nematode groups, depth layers (δ^13^C averaged over two 1 cm slices) and treatments. The resulting SF is a quantitative measure (0–100%) of labelled diatom selection by the nematodes. A SF-value <0 results from a sedimentary δTO^13^C higher than the δ^13^C of nematodes in a specific depth layer. This could either be due to the fact that steady-state was not reached or point to nematodes feeding non-selectively in layers where the organic matter is less labelled, and later migrating to the layer where they were sampled. Since it is highly unlikely that steady state was not reached after 18 days, a SF-value <0 is ascribed to migration.

To test the difference in profiles of nematode densities and environmental variables, non-parametric permutational ANOVAs (Permanova) with a fully crossed three-factor design were performed with random factor replicate [Rep] nested in the fixed factor treatment [TR], next to the fixed factor depth [D]. The interaction term TRxD informs about the difference in depth profiles of nematode densities or environmental variables among treatments. Since data from different depth layers from a single replicate core are not fully independent, we chose to run these permutational ANOVAs; they can be used as univariate ANOVAs with p-values obtained by permutation [55]. A Euclidean distance based resemblance matrix was used. In case of significant TRxD interactions, pairwise tests of TR and D within TRxD were performed to investigate in which slice the treatments differed or vice versa. Because of the restricted number of possible permutations in pairwise tests, p-values were obtained from Monte Carlo samplings [56]. To test the difference in uptake of algae by nematodes (Δδ^13^C and total uptake) among treatments, depth layers and nematode groups, a fully crossed 4-factor Permanova was carried out with TR, D and nematode group [Sp] as fixed factors and Rep nested in TR. Homogeneity of multivariate dispersion was confirmed by Permdisp for any of the tested terms in each Permanova which indicates that patterns found were not confounded by artefacts due to variable dispersions. Again pairwise tests were carried out when factors interacted significantly. Difference in remaining chl-*a* and TO^13^C in the sediment, oxygen penetration depth and nematode survival were tested with a single factor (TR) Permanova with subsequent pairwise tests. A two-factor Permanova was carried out to test the difference in oxygen consumption among TR and measurement day. All analyses were performed within PRIMER v6 with PERMANOVA+ add-on software [57], [58]. Results are expressed as mean values ± standard error of triplicates, except for the C treatment that is represented by mean values of duplicates and their standard deviation.

## Supporting Information

Table S1 **Results from Permanova analysis: Pair wise tests of TR within TRxD for differences in Chlorophyll-** ***a*** **amongst experimental treatments and depth, based on a normalised Euclidean resemblance matrix.** The significantly different depths among treatments are indicated with p-values drawn from Monte-Carlo samplings.
(DOCX)

Table S2 **Results from Permanova analysis: Pair wise tests of D within TRxD for differences in Chlorophyll-** ***a*** **amongst experimental treatments and depth, based on a normalised Euclidean resemblance matrix.** The significantly different depths within treatments are indicated with p-values drawn from Monte-Carlo samplings.
(DOCX)

Table S3 **Results from Permanova analysis: Pair wise tests of TR within TRxD for differences in total TO^13^C within the sediment amongst experimental treatments and depth, based on a normalised Euclidean resemblance matrix.** The significantly different depths among treatments are indicated with p-values drawn from Monte-Carlo samplings.
(DOCX)

Table S4 **Results from Permanova analysis: Pair wise tests of D within TRxD for differences in total TO^13^C within the sediment amongst experimental treatments and depth, based on a normalised Euclidean resemblance matrix.** The significantly different depths within treatments are indicated with p-values drawn from Monte-Carlo samplings.
(DOCX)

Table S5 **Results from Permanova analysis: Pair wise tests of oxygen penetration depth amongst experimental treatments, based on a normalised Euclidean resemblance matrix.** The significantly different treatments are indicated with p-values drawn from Monte-Carlo samplings.
(DOCX)

Table S6 **Results from Permanova analysis: Pair wise tests of TR within TRxD for differences in nematode density (ind. 10 cm^−2^) amongst experimental treatments and depth, based on a normalised Euclidean resemblance matrix.** The significantly different depths among treatments are indicated with p-values drawn from Monte-Carlo samplings.
(DOCX)

Table S7 **Results from Permanova analysis: Pair wise tests of D within TRxD for differences in nematode density (ind. 10 cm^−2^) amongst experimental treatments and depth, based on a normalised Euclidean resemblance matrix.** The significantly different depths among treatments are indicated with p-values drawn from Monte-Carlo samplings.
(DOCX)
