# Supplementary material for: Biological vs. Physical Mixing Effects on Benthic Food Web Dynamics
Source: PLoS One. 2011 Mar 24;6(3):e18078. doi: 10.1371/journal.pone.0018078 (PMC3063793; doi:10.1371/journal.pone.0018078)
Supplement: Table S1 — Results from Permanova analysis: Pair wise tests of TR within TRxD for differences in Chlorophyll- a amongst experimental treatments and depth, based on a normalised Euclidean resemblance matrix. The significantly different depths among treatments are indicated with p-values drawn from Monte-Carlo samplings. (DOCX) [file pone.0018078.s001.docx]

Table S1

| *Depth (cm)* | *Groups* | *t* | *P(MC)* |
| --- | --- | --- | --- |
| 0-1 | PM, BT | 7.31 | 0.002 |
|  | PM, CF | 3.58 | 0.026 |
|  | PM, C | 6.68 | 0.006 |
|  | BT, CF | 8.59 | 0.001 |
|  | BT, BI | 3.87 | 0.018 |
|  | BT, C | 10.32 | 0.002 |
| 1-2 | PM, BT | 3.33 | 0.029 |
|  | BT, CF | 3.03 | 0.037 |
| 4-5 | BT, BI | 4.30 | 0.012 |
|  | BI, C | 4.31 | 0.025 |
