# Supplementary material for: Biological vs. Physical Mixing Effects on Benthic Food Web Dynamics
Source: PLoS One. 2011 Mar 24;6(3):e18078. doi: 10.1371/journal.pone.0018078 (PMC3063793; doi:10.1371/journal.pone.0018078)
Supplement: Table S2 — Results from Permanova analysis: Pair wise tests of D within TRxD for differences in Chlorophyll- a amongst experimental treatments and depth, based on a normalised Euclidean resemblance matrix. The significantly different depths within treatments are indicated with p-values drawn from Monte-Carlo samplings. (DOCX) [file pone.0018078.s002.docx]

Table S2

| *Depth groups* | *t* | *P(MC)* |  | *Depth groups* | *t* | *P(MC)* |  | *Depth groups* | *t* | *P(MC)* |
| --- | --- | --- | --- | --- | --- | --- | --- | --- | --- | --- |
| **C** |  |  |  | **BT** |  |  |  | **PM** |  |  |
| 0-1, 2-3 | 176.03 | **0.004** |  | 0-1, 1-2 | 14.60 | **0.005** |  | 0-1, 2-3 | 6.48 | **0.022** |
| 0-1, 3-4 | 18.25 | **0.036** |  | 0-1, 5-6 | 5.13 | **0.036** |  | 0-1, 3-4 | 12.08 | **0.007** |
| 0-1, 4-5 | 40.84 | **0.016** |  | 0-1, 6-7 | 6.87 | **0.019** |  | 0-1, 4-5 | 33.34 | **0.001** |
| 0-1, 5-6 | 45.64 | **0.014** |  | 0-1, 8-9 | 4.81 | **0.042** |  | 0-1, 5-6 | 15.74 | **0.004** |
| 0-1, 6-7 | 61.27 | **0.010** |  | 1-2, 3-4 | 5.97 | **0.030** |  | 0-1, 6-7 | 15.59 | **0.005** |
| 0-1, 7-8 | 35.46 | **0.018** |  | 1-2, 4-5 | 5.47 | **0.032** |  | 0-1, 7-8 | 26.65 | **0.001** |
| 5-6, 6-7 | 15.64 | **0.042** |  | 1-2, 5-6 | 9.60 | **0.010** |  | 0-1, 8-9 | 24.73 | **0.002** |
| **CF** |  |  |  | 1-2, 6-7 | 14.42 | **0.004** |  | 1-2, 3-4 | 30.66 | **0.002** |
| 0-1, 1-2 | 6.05 | **0.027** |  | 1-2, 7-8 | 6.82 | **0.022** |  | 1-2, 4-5 | 28.34 | **0.001** |
| 0-1, 2-3 | 7.01 | **0.020** |  | 1-2, 8-9 | 7.78 | **0.016** |  | 1-2, 5-6 | 7.05 | **0.020** |
| 0-1, 3-4 | 9.13 | **0.011** |  | 2-3, 4-5 | 16.36 | **0.005** |  | 1-2, 6-7 | 8.35 | **0.014** |
| 0-1, 4-5 | 9.13 | **0.012** |  | 2-3, 5-6 | 36.87 | **0.001** |  | 1-2, 7-8 | 9.94 | **0.010** |
| 0-1, 5-6 | 11.06 | **0.009** |  | 2-3, 6-7 | 9.26 | **0.012** |  | 1-2, 8-9 | 9.93 | **0.009** |
| 0-1, 6-7 | 12.41 | **0.006** |  | 2-3, 7-8 | 17.83 | **0.003** |  | 2-3, 4-5 | 4.76 | **0.044** |
| 0-1, 7-8 | 10.27 | **0.009** |  | 2-3, 8-9 | 20.47 | **0.003** |  | 2-3, 5-6 | 4.67 | **0.042** |
| 0-1, 8-9 | 22.35 | **0.002** |  | 3-4, 5-6 | 4.68 | **0.042** |  | 2-3, 6-7 | 17.71 | **0.002** |
| 1-2, 2-3 | 8.22 | **0.015** |  | 4-5, 7-8 | 13.89 | **0.006** |  | 2-3, 7-8 | 6.17 | **0.026** |
| 1-2, 3-4 | 10.70 | **0.008** |  | 4-5, 8-9 | 8.34 | **0.015** |  | 2-3, 8-9 | 6.11 | **0.025** |
| 1-2, 4-5 | 19.19 | **0.003** |  | **BI** |  |  |  | 5-6, 7-8 | 5.69 | **0.027** |
| 1-2, 5-6 | 63.91 | **0.000** |  | 0-1, 4-5 | 5.16 | **0.040** |  | 5-6, 8-9 | 7.36 | **0.018** |
| 1-2, 6-7 | 24.15 | **0.001** |  | 0-1, 5-6 | 4.53 | **0.047** |  | 7-8, 8-9 | 5.18 | **0.036** |
| 1-2, 7-8 | 19.19 | **0.003** |  | 0-1, 6-7 | 4.77 | **0.039** |  |  |  |  |
| 1-2, 8-9 | 11.22 | **0.006** |  | 0-1, 7-8 | 4.49 | **0.047** |  |  |  |  |
| 2-3, 3-4 | 10.63 | **0.010** |  | 0-1, 8-9 | 5.28 | **0.031** |  |  |  |  |
| 2-3, 4-5 | 7.61 | **0.015** |  |  |  |  |  |  |  |  |
| 2-3, 5-6 | 8.14 | **0.018** |  |  |  |  |  |  |  |  |
| 2-3, 6-7 | 14.89 | **0.004** |  |  |  |  |  |  |  |  |
| 2-3, 7-8 | 6.56 | **0.021** |  |  |  |  |  |  |  |  |
| 3-4, 6-7 | 4.52 | **0.045** |  |  |  |  |  |  |  |  |
