# Supplementary material for: Biological vs. Physical Mixing Effects on Benthic Food Web Dynamics
Source: PLoS One. 2011 Mar 24;6(3):e18078. doi: 10.1371/journal.pone.0018078 (PMC3063793; doi:10.1371/journal.pone.0018078)
Supplement: Table S3 — Results from Permanova analysis: Pair wise tests of TR within TRxD for differences in total TO13C within the sediment amongst experimental treatments and depth, based on a normalised Euclidean resemblance matrix. The significantly different depths among treatments are indicated with p-values drawn from Monte-Carlo samplings. (DOCX) [file pone.0018078.s003.docx]

Table S3

| *Depth (cm)* | *Groups* | *t* | *P(MC)* |
| --- | --- | --- | --- |
| 1-2 | BI, PM | 5.38 | **0.005** |
|  | BI, BT | 5.40 | **0.006** |
|  | PM, BT | 4.46 | **0.011** |
|  | PM, CF | 5.41 | **0.005** |
|  | BT, CF | 5.87 | **0.004** |
| 2-3 | BI, BT | 3.55 | **0.025** |
| 5-6 | PM, CF | 2.85 | **0.046** |
| 6-7 | BI, PM | 3.19 | **0.033** |
|  | BI, BT | 3.51 | **0.024** |
| 7-8 | BI, PM | 3.31 | **0.025** |
|  | BI, BT | 3.51 | **0.025** |
|  | BI, CF | 3.20 | **0.032** |
| 8-9 | BI, PM | 3.31 | **0.025** |
|  | BI, BT | 3.51 | **0.025** |
|  | BI, CF | 3.20 | **0.032** |
