# Supplementary material for: Biological vs. Physical Mixing Effects on Benthic Food Web Dynamics
Source: PLoS One. 2011 Mar 24;6(3):e18078. doi: 10.1371/journal.pone.0018078 (PMC3063793; doi:10.1371/journal.pone.0018078)
Supplement: Table S4 — Results from Permanova analysis: Pair wise tests of D within TRxD for differences in total TO13C within the sediment amongst experimental treatments and depth, based on a normalised Euclidean resemblance matrix. The significantly different depths within treatments are indicated with p-values drawn from Monte-Carlo samplings. (DOCX) [file pone.0018078.s004.docx]

Table S4

| *Depth groups* | *t* | *P(MC)* |  | *Depth groups* | *t* | *P(MC)* |  | *Depth groups* | *t* | *P(MC)* |
| --- | --- | --- | --- | --- | --- | --- | --- | --- | --- | --- |
| **CF** |  |  |  | **BT** |  |  |  | **PM** |  |  |
| 0-1, 2-3 | 4.53 | **0.046** |  | 0-1, 1-2 | 9.93 | **0.011** |  | 0-1, 2-3 | 6.61 | **0.022** |
| 0-1, 4-5 | 4.39 | **0.049** |  | 0-1, 2-3 | 9.89 | **0.009** |  | 0-1, 3-4 | 6.51 | **0.028** |
| 0-1, 5-6 | 4.28 | **0.049** |  | 0-1, 3-4 | 9.60 | **0.011** |  | 0-1, 4-5 | 6.31 | **0.025** |
| 0-1, 6-7 | 4.42 | **0.043** |  | 0-1, 4-5 | 9.49 | **0.012** |  | 0-1, 5-6 | 6.43 | **0.023** |
| 0-1, 7-8 | 4.45 | **0.046** |  | 0-1, 5-6 | 8.05 | **0.016** |  | 0-1, 6-7 | 6.47 | **0.024** |
| 1-2, 3-4 | 4.54 | **0.049** |  | 0-1, 6-7 | 9.76 | **0.010** |  | 0-1, 7-8 | 6.50 | **0.023** |
| 1-2, 4-5 | 7.11 | **0.020** |  | 0-1, 7-8 | 9.60 | **0.010** |  | 0-1, 8-9 | 6.50 | **0.022** |
| 1-2, 7-8 | 7.41 | **0.019** |  | 0-1, 8-9 | 9.41 | **0.012** |  | 1-2, 2-3 | 7.26 | **0.018** |
| 1-2, 8-9 | 11.06 | **0.007** |  | 1-2, 2-3 | 7.12 | **0.020** |  | 1-2, 3-4 | 5.65 | **0.031** |
| 3-4, 4-5 | 11.92 | **0.006** |  | 1-2, 3-4 | 6.74 | **0.022** |  | 1-2, 4-5 | 5.57 | **0.029** |
| 4-5, 5-6 | 7.22 | **0.019** |  | 1-2, 4-5 | 7.25 | **0.018** |  | 1-2, 5-6 | 5.67 | **0.032** |
| **BI** |  |  |  | 1-2, 6-7 | 7.66 | **0.017** |  | 1-2, 6-7 | 5.70 | **0.031** |
| 0-1, 1-2 | 5.38 | **0.033** |  | 1-2, 7-8 | 7.51 | **0.019** |  | 1-2, 7-8 | 5.68 | **0.030** |
| 0-1, 2-3 | 5.13 | **0.035** |  | 1-2, 8-9 | 6.24 | **0.026** |  | 1-2, 8-9 | 5.68 | **0.028** |
| 0-1, 3-4 | 5.51 | **0.032** |  | 2-3, 3-4 | 5.22 | **0.039** |  | 3-4, 7-8 | 5.63 | **0.026** |
| 0-1, 4-5 | 4.64 | **0.040** |  | 2-3, 4-5 | 4.82 | **0.041** |  | 3-4, 8-9 | 5.69 | **0.029** |
| 0-1, 5-6 | 4.92 | **0.037** |  | 2-3, 6-7 | 7.64 | **0.018** |  | 7-8, 8-9 | 5.10 | **0.035** |
|  |  |  |  | 2-3, 7-8 | 5.71 | **0.031** |  |  |  |  |
