# Supplementary material for: Biological vs. Physical Mixing Effects on Benthic Food Web Dynamics
Source: PLoS One. 2011 Mar 24;6(3):e18078. doi: 10.1371/journal.pone.0018078 (PMC3063793; doi:10.1371/journal.pone.0018078)
Supplement: Table S5 — Results from Permanova analysis: Pair wise tests of oxygen penetration depth amongst experimental treatments, based on a normalised Euclidean resemblance matrix. The significantly different treatments are indicated with p-values drawn from Monte-Carlo samplings. (DOCX) [file pone.0018078.s005.docx]

Table S5

| *TR groups* | *t* | *P(MC)* |
| --- | --- | --- |
| C, BI | 3.25 | 0.006 |
| C, PM | 7.22 | 0.001 |
| CF, BI | 3.14 | 0.006 |
| CF, PM | 5.02 | 0.001 |
| BT, BI | 3.84 | 0.001 |
| BT, PM | 2.22 | 0.028 |
| BI, PM | 5.55 | 0.001 |
