# Supplementary material for: Biological vs. Physical Mixing Effects on Benthic Food Web Dynamics
Source: PLoS One. 2011 Mar 24;6(3):e18078. doi: 10.1371/journal.pone.0018078 (PMC3063793; doi:10.1371/journal.pone.0018078)
Supplement: Table S6 — Results from Permanova analysis: Pair wise tests of TR within TRxD for differences in nematode density (ind. 10 cm−2) amongst experimental treatments and depth, based on a normalised Euclidean resemblance matrix. The significantly different depths among treatments are indicated with p-values drawn from Monte-Carlo samplings. (DOCX) [file pone.0018078.s006.docx]

Table S6

| *Slice (cm)* | *TR groups* | *t* | *P(MC)* |
| --- | --- | --- | --- |
| 0-1 | BT, C | 3.78 | **0.030** |
|  | PM, C | 11.98 | **0.001** |
| 2-3 | CF, BI | 7.95 | **0.002** |
|  | CF, PM | 10.42 | **0.001** |
|  | BI, PM | 4.30 | **0.012** |
|  | BI, C | 10.99 | **0.002** |
|  | PM, C | 11.44 | **0.002** |
| 3-4 | CF, BI | 4.97 | **0.008** |
|  | CF, PM | 4.37 | **0.013** |
| 4-5 | CF, BI | 5.56 | **0.006** |
|  | BT, BI | 7.10 | **0.002** |
|  | BI, PM | 2.92 | **0.044** |
|  | BI, C | 4.34 | **0.024** |
| 5-6 | CF, BI | 7.86 | **0.002** |
|  | CF, PM | 8.27 | **0.001** |
|  | BT, BI | 9.26 | **0.001** |
|  | BT, PM | 14.09 | **<0.001** |
|  | BI, PM | 4.31 | **0.013** |
|  | BI, C | 5.37 | **0.011** |
|  | PM, C | 4.49 | **0.022** |
| 6-7 | CF, BI | 10.05 | **0.001** |
|  | BT, BI | 11.04 | **<0.001** |
|  | BI, PM | 6.93 | **0.002** |
|  | BI, C | 11.40 | **0.001** |
