# Supplementary material for: Biological vs. Physical Mixing Effects on Benthic Food Web Dynamics
Source: PLoS One. 2011 Mar 24;6(3):e18078. doi: 10.1371/journal.pone.0018078 (PMC3063793; doi:10.1371/journal.pone.0018078)
Supplement: Table S7 — Results from Permanova analysis: Pair wise tests of D within TRxD for differences in nematode density (ind. 10 cm−2) amongst experimental treatments and depth, based on a normalised Euclidean resemblance matrix. The significantly different depths among treatments are indicated with p-values drawn from Monte-Carlo samplings. (DOCX) [file pone.0018078.s007.docx]

Table S7

| *Depth groups* | *t* | *P(MC)* |  | *Depth groups* | *t* | *P(MC)* |  | *Depth groups* | *t* | *P(MC)* |
| --- | --- | --- | --- | --- | --- | --- | --- | --- | --- | --- |
| **C** |  |  |  | **BT** |  |  |  | **PM** |  |  |
| 0-1, 5-6 | 20.23 | **0.034** |  | 1-2, 3-4 | 6.15 | **0.026** |  | 0-1, 1-2 | 5.54 | **0.032** |
| 0-1, 6-7 | 13.38 | **0.046** |  | 1-2, 4-5 | 4.37 | **0.049** |  | 0-1, 2-3 | 4.72 | **0.042** |
| 0-1, 7-8 | 14.47 | **0.044** |  | 1-2, 5-6 | 4.39 | **0.048** |  | 0-1, 5-6 | 4.67 | **0.046** |
| 1-2, 3-4 | 103.00 | **0.006** |  | 1-2, 7-8 | 4.42 | **0.049** |  | 0-1, 6-7 | 5.64 | **0.030** |
| 2-3, 6-7 | 21.00 | **0.029** |  | 4-5, 5-6 | 5.00 | **0.040** |  | 0-1, 7-8 | 7.04 | **0.022** |
| **CF** |  |  |  | **BI** |  |  |  | 0-1, 8-9 | 27.53 | **0.002** |
| 1-2, 3-4 | 5.50 | **0.034** |  | 3-4, 8-9 | 7.57 | **0.016** |  | 1-2, 5-6 | 5.07 | **0.035** |
| 1-2, 4-5 | 4.95 | **0.039** |  |  |  |  |  | 1-2, 6-7 | 5.63 | **0.032** |
| 1-2, 5-6 | 6.96 | **0.018** |  |  |  |  |  | 1-2, 7-8 | 6.63 | **0.023** |
| 1-2, 6-7 | 6.80 | **0.023** |  |  |  |  |  | 1-2, 8-9 | 18.57 | **0.004** |
| 1-2, 7-8 | 5.81 | **0.028** |  |  |  |  |  | 2-3, 3-4 | 4.57 | **0.044** |
| 1-2, 8-9 | 5.35 | **0.033** |  |  |  |  |  | 2-3, 4-5 | 9.61 | **0.012** |
| 2-3,3-4 | 13.00 | **0.006** |  |  |  |  |  | 2-3, 5-6 | 21.36 | **0.002** |
| 2-3, 5-6 | 4.56 | **0.044** |  |  |  |  |  | 2-3, 6-7 | 53.00 | **0.000** |
| 2-3, 6-7 | 24.57 | **0.001** |  |  |  |  |  | 2-3, 7-8 | 28.38 | **0.001** |
| 2-3, 7-8 | 7.94 | **0.016** |  |  |  |  |  | 2-3, 8-9 | 11.88 | **0.008** |
| 3-4, 6-7 | 7.21 | **0.019** |  |  |  |  |  | 3-4, 6-7 | 5.36 | **0.032** |
| 4-5, 8-9 | 6.93 | **0.023** |  |  |  |  |  | 3-4, 8-9 | 5.68 | **0.029** |
|  |  |  |  |  |  |  |  | 5-6, 6-7 | 7.01 | **0.020** |
|  |  |  |  |  |  |  |  | 5-6, 7-8 | 5.96 | **0.025** |
|  |  |  |  |  |  |  |  | 5-6, 8-9 | 6.50 | **0.022** |
